# Interaction between Nitrogen and Sulfur in Co-Doped Graphene and Synergetic Effect in Supercapacitor

**DOI:** 10.1038/srep09591

**Published:** 2015-04-16

**Authors:** Tao Wang, Lu-Xiang Wang, Dong-Ling Wu, Wei Xia, Dian-Zeng Jia

**Affiliations:** 1 Key Laboratory of Material and Technology for Clean Energy, Ministry of Education, Key Laboratory of Advanced Functional Materials, Autonomous Region, Institute of Applied Chemistry, Xinjiang University, 830046 Xinjiang, P. R. China; 2 Laboratory of Industrial Chemistry, Ruhr-University Bochum, 44780 Bochum, Germany

## Abstract

The co-doping of graphene with nitrogen and sulfur was investigated aiming at understanding their interactions with the presence of oxygen in graphene. The co-doped graphene (NS-G) was synthesized via a one-pot hydrothermal route using graphene oxide as starting material and L-cysteine, an amino acid containing both N and S, as the doping agent. The obtained NS-G with a three-dimensional hierarchical structure containing both macropores and mesopores exhibited excellent mechanical stabilities under both wet and dry conditions. As compared to N or S singly doped graphene, the co-doped sample contains significantly higher concentrations of N and S species especially pyrollic N groups. The co-doped sample considerably outperformed the singly doped samples when used as free-standing electrode in supercapacitors due to enhanced pseudocapacitance. The simultaneous incorporation of S and N species with the presence of oxygen significantly modified the surface chemistry of carbon leading to considerably higher doping levels, although directly bonding between N and S is neither likely nor detected. Hence, the synergetic effect between N and S occurred through carbon atoms in neighboring hexagonal rings in a graphene sheet.

The incorporation of heteroatoms in carbon materials can significantly modify their surface and bulk properties and considerably broaden their applications in various fields. Among others, nitrogen doping of graphene has been intensively investigated in the last few years. Sulfur and boron are among other commonly used heteroatoms for the doping^1^,^2^. Chemically obtained graphene oxide (GO) or graphene inevitably contains oxygen, and the nitrogen doping can thus be realized by partially replacing oxygen with nitrogen through wet chemistry method or thermal treatment in the gas phase. The co-doping of graphene with nitrogen and sulfur is therefore even more complicated due to the involvement of oxygen, let alone the interference from defects. Hence, the chemistry involved therein is far from understood.

Recently, graphene has been widely explored as electrode materials in supercapacitors due to its large specific surface area and high electronic conductivity^3^,^4^,^5^,^6^,^7^. The capacitances of graphene-based electrochemical double-layer capacitors are typically in the range of 100–200 F g^−1^, which are far below the theoretical electrochemical double-layer capacitance of 520 F g^−1^^8^. There are two basic and usually complementary approaches by which the properties of graphene can be effectively regulated: the design of diverse morphologies and the incorporation of certain heteroatoms in the carbon matrix^9^,^10^.

Much progress has been made in the synthesis of graphene with different morphologies so far. For example, two-dimensional graphene nanosheets, one-dimensional graphene nanoribbons, and zero-dimensional graphene quantum dots have been obtained^9^,^11^,^12^,^13^. Moreover, activated graphene^14^, graphene-assembled foam^15^, graphene paper^16^, graphene fiber^17^, graphene-based hydrogel or aerogel^18^,^19^ and nanoporous graphene^20^,^21^ materials have been synthesized by different methods, and the obtained materials were used to prepare macroscopic, large dimension monolith for further applications. As a result, the performance of graphene-based materials used as electrode in supercapacitors has been improved^4^,^5^,^14^,^15^,^16^,^17^,^18^,^19^,^20^.

Other than the morphology control, another effective way in improving the capacitive performance of graphene is the introduction of heteroatoms^22^,^23^. Recent progress demonstrated that substitutional doping of graphene with heteroatoms could substantially modify the electrical conductivity, surface activity, chemical reactivity and mechanical properties of graphene, which are essential for energy storage applications^24^,^25^,^26^.

It has been reported that nitrogen doping can improve the specific capacitance and cyclability of graphene in supercapacitors^14^,^27^,^28^,^29^. Furthermore, comparing with singly doping, multiple doping is a versatile synthetic approach, which can further tune the properties of monodoped graphene. Usually, nitrogen doping is preferential in tuning the electronic properties of the carbon material, whereas sulfur, due to its larger size, has been used for applications where its easily polarizable electron pairs and thus higher chemical reactivity are of interest^30^.

Although co-doped carbon materials potentially possess promising properties for various applications, there are only a few reports about graphene that are simultaneously doped with sulfur and nitrogen^31^,^32^,^33^,^34^,^35^,^36^,^37^. Several nitrogen and sulfur sources were involved for the co-doping including melamine and benzyl disulfide^31^, thiourea^32^,^33^, pyrimidine and thiophene^34^, bacteria^35^, PVP and sulfonated polystyrene^36^, and ammonium thiocyanate^37^. The nitrogen and sulfur co-doped graphene (NS-G) were used in electrocatalytic oxygen reduction reaction and in lithium ion battery and showed promising performance. To our knowledge, the application of NS-G in supercapacitors has not been reported so far.

Recently, we used GO as precursor and nine different amino acids as environment-friendly nitrogen sources for the synthesis of nitrogen-doped graphene via hydrothermal method, where the influence of acidity of amino acid on the capacitive performance was investigated. The charged amino acid, and the ensuing electrostatic interactions between amino acid and GO affect the morphology and specific surface area of nitrogen doped graphene (N-G), and ultimately affect its electrochemical performance. The specific capacitance for the obtained N-G samples are following the order of acidic amino acids > neutral amino acids > basic amino acids^38^.

The present study aims at the understanding of the interaction between nitrogen and sulfur as dopants in graphene. Graphene oxide and L-cysteine were used as starting material in the hydrothermal synthesis. The obtained NS-G exhibits three-dimensional hierarchical structure containing macropores. The NS-G hydrogel with high mechanical stabilities was used as an additive/binder-free electrode and showed an excellent electrochemical performance with a high specific capacitance of 566 F g^−1^, an energy density of 29.4 Wh kg^−1^ and a power density of 10.0 kW kg^−1^, which significantly outperformed the nitrogen doped graphene (N-G) and sulfur doped graphene (S-G). The location, interaction and synergetic effect of nitrogen and sulfur on carbon surface were discussed.

## Results

**Figure 1:**
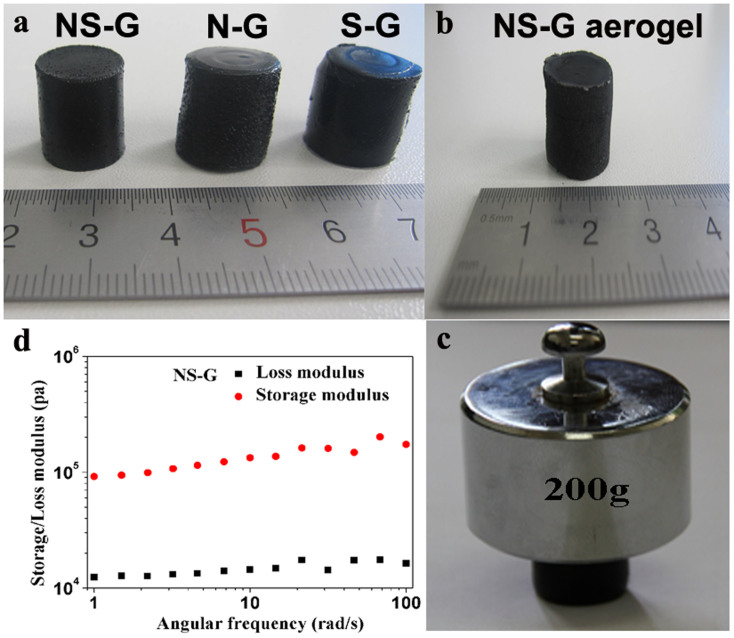
Photographs of the doped samples and the mechanical performance of NS-G. (a) The prepared NS-G, N-G and S-G hydrogels. (b) NS-G aerogel obtained by freeze-drying. (c) NS-G hydrogel can sustain 200 g weight loading. (d) Mechanical property of the obtained NS-G hydrogels.

The co-doped NS-G, singly doped N-G and S-G hydrogels were successfully synthesized via the one-pot hydrothermal method. When preparing using the same reactor, the dimension of the hydrogel with different doping elements appears to be rather similar (Figure 1a). We also performed the synthesis with different reactors, and the concentration and reaction time were also varied. It was found that the dimension and form of the doped hydrogels could be easily adjusted by varying the concentration of GO, reaction time and the volume of autoclave. By weighing the samples before and after drying it was found that the obtained NS-G, S-G and N-G hydrogels contained about 97.6 wt%, 98.7 wt% and 98.1 wt% of water, respectively. Freeze-drying did not lead to visible volume contraction of the hydrogel and the dimension of the obtained areogel remained similar (Figure 1b). However, the mechanical stability of these hydrogel samples showed clear differences as disclosed by loading tests. The N-G and S-G samples crushed when 100 g weight was loaded (not shown), whereas the NS-G hydrogel of the same dimension sustained 200 g without visible deformation (Figure 1c, d). Sufficient mechanical stability is essential for electrode materials and the high mechanical stability of the co-doped hydrogel allows it being used as free-standing electrode. More detailed evaluation of the mechanical property of the NS-G hydrogel was performed by small-deformation oscillatory measurement. As can be seen from Figure 1d, the storage modulus (G′) of NS-G is about 10 times than that of its loss modulus (G″) in the frequency range of 1–100 rad s^−1^, indicating a predominantly elastic response. The G″ of NS-G hydrogel is slightly sensitive to the frequency and does not cross over G′, implying a high degree of covalent or non-covalent cross-linking in the hydrogel^13^,^18^. Moreover, the G′ value of the NS-G hydrogel was determined to be about 135 kPa at 10 rad/s, which is much higher than the doped graphene hydrogel^19^ and about 1-2 orders of magnitude higher than those of conventional self-assembled hydrogels^18^. The high mechanical stability of the co-doped hydrogel points to its high potential as free-standing electrode in practical applications.

**Figure 2:**
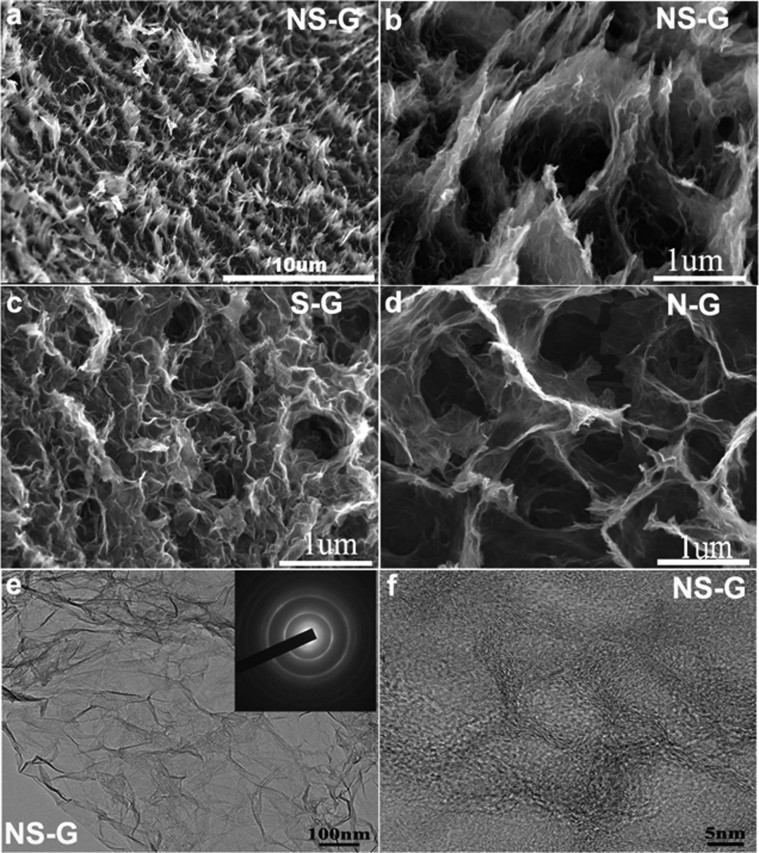
Morphological characterization of the doped graphene. (a) and (b) SEM images of NS-G at low and high magnifications. (c) and (d) SEM images of S-G and N-G aerogel respectively. (e) and (f) TEM images of NS-G. Inset of (e) is the corresponding electron diffraction pattern.

The morphology of the NS-G, S-G and N-G aerogel samples was investigated by SEM. As displayed in Figure 2a, the obtained NS-G appears to be an interconnected network with uniform mesoporous structure at low magnification. Remarkably, the mesoporous network of NS-G shows a very loose structure at high magnification, and the pore size ranges from submicrometer to several micrometers (Figure 2b). SEM studies disclosed that the co-doped samples prepared from different cysteine to GO ratios, i.e., NS-G005 and NS-G1, showed similar loose 3D structures (Figure S1). Similar morphology can be observed for S-G and N-G (Figure 2c, d). It is believed that the 3D porous network can contribute to the fast adsorption and diffusion of the ions from the electrolyte, which can enhance the electrochemical performance when used as electrode in supercapacitors. TEM studies disclosed a laminar morphology with abundant wrinkles (Figure 2e). Electron diffraction measurements yielded ring-like pattern indicating that N and S dopants introduced a significant amount of structural irregularity into the hexagonal crystal lattice of graphene. High-resolution TEM was carried out to investigate the detailed structure of the NS-G. As shown in Figure 2f, a set of highly interconnected crumpled wrinkles with continuous pores structure can be seen, indicating that these pores are caused by the crumpling of graphene.

**Figure 3:**
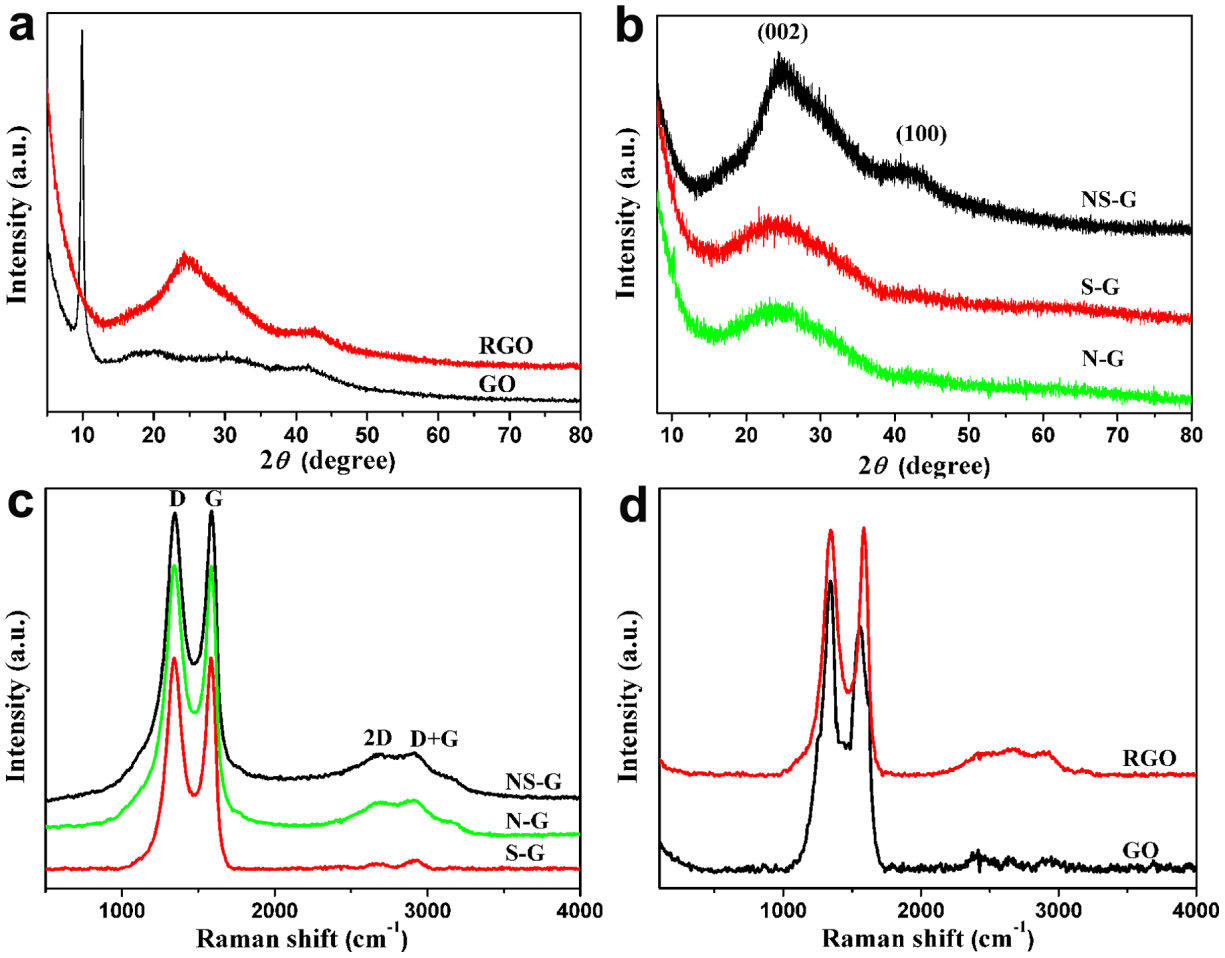
XRD and Raman characterization. XRD patterns of (a) GO and RGO, (b) NS-G, S-G and N-G respectively. Raman spectra of (c) NS-G, S-G and N-G, (d) GO and RGO.

The results from XRD measurements are shown in Figure 3a and b. The diffraction peak of GO at 2θ = 9.9° can be attributed to the (002) crystalline plane (Figure 3a), and the calculated interlayer spacing is about 0.89 nm. This peak entirely disappeared after the hydrothermal reaction. Instead, broad contributions appeared in the freeze-dried RGO (Figure 3a) and doped graphene (Figure 3b), indicating the recovery of π-conjugated system from GO sheets under hydrothermal reaction. As compared to NS-G, the broad and weak contributions of the (002) and (100) in S-G and N-G indicate the poorly ordered graphene sheets along their stacking direction. Using the Bragg equation, the interlayer spacing of NS-G, S-G and N-G was calculated to be 0.36, 0.37 and 0.37 nm, respectively. These values are much lower than that of GO (0.89 nm) but slightly higher than that of graphite (0.335 nm), indicating the recovery of a graphitic crystal structure after hydrothermal reaction.

Figure 3c and d show the Raman spectra of the prepared samples. All the spectra exhibit two main peaks at about 1340 and 1580 cm^−1^ corresponding to the D and G band, respectively (Figure 3c). The relative intensities between D band and G band of NS-G, N-G and S-G appear to be lower than that of GO (Figure 3c and d), indicating the recovery of the conjugated network in graphene to certain extent. Furthermore, the I_D_/I_G_ values of NS-G (0.99), S-G (1.03) and N-G (1.00) are essentially equal, which means the defect levels of these samples are similar. In the doped samples, the weak and broad peaks at around 2800 cm^−1^ can be assigned to the combination of 2D-band and D+G-band. The peaks at around 2680 cm^−1^ are related to the second-order two phonon mode 2D-band and the weak contribution at around 2920 cm^−1^ is a combination of the D-and G-band, which are related to the disorder-induced feature of carbon material^39^. In addition, The Raman spectrum of RGO was measured and shown in Figure 3d. A weak and broad peak at around 3000 cm^−1^ is also observed. The presence of the broad peak in RGO, NS-G, N-G and S-G confirms the presence of defects.

**Figure 4:**
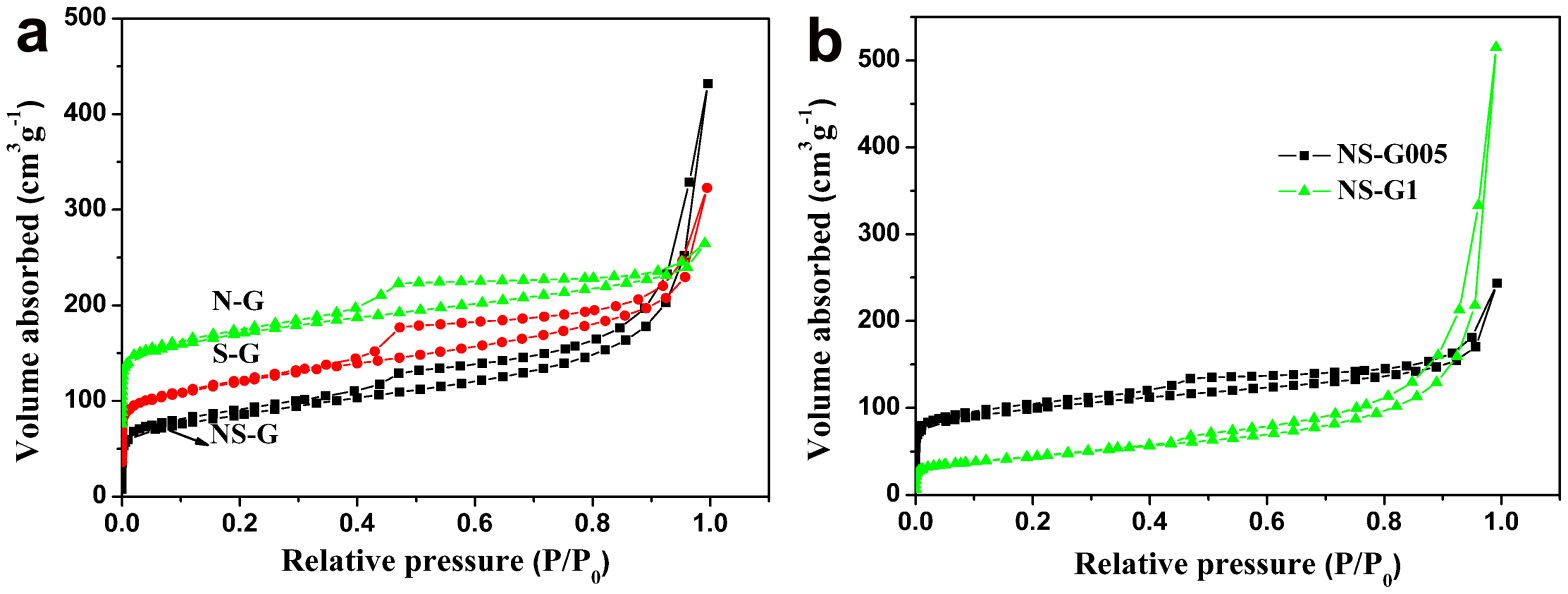
surface area characterization. N_2_ adsorption-desorption isotherms of (a) NS-G, S-G, N-G, (b) NS-G005 and NS-G1.

The BET surface area and pore structure of the freeze-dried samples were investigated by nitrogen physisorption, and the obtained isotherms are shown in Figure 4. It can be seen that the isotherms of NS-G, S-G and N-G are characteristic of the type IV shape (Figure 4a) with a distinct hysteresis loop in the medium to high pressure regions (P/P_0_ = 0.4-1). The co-doped samples prepared from different cysteine to GO ratios show similar pore structure but with less pronounced hysteresis in isotherms (Figure 4b). As a whole, it can be concluded that all samples have typically mesoporous structure. The BET surface area, pore size and pore volume of NS-G, S-G and N-G are summarized in Table 1. The BET surface area of NS-G (305.2 m^2^ g^−1^) is slightly lower than those of S-G and N-G. It is also lower than the nitrogen-doped graphene prepared using acidic amino acid in our previously study (367.1 m^2^ g^−1^)^38^. Notably, the specific surface of the NS-G is much higher than those reported for sulfur and nitrogen co-doped graphene materials, which are typically in the range of 70 to 220 m^2^ g^−1^^29^,^33^,^40^. Moreover, the specific surface of the NS-G aerogel is much higher than co-doped samples synthesized using other ratios (285.1 m^2^ g^−1^ for NS-G005 and 156.9 m^2^ g^−1^ for NS-G1).

The pore size distributions are listed in Table 1. The NS-G areogel exhibits the largest pore size of 14.8 nm with a total pore volume of 0.61 cm^3^ g^−1^. This is considered as a positive feature in this work, since mesoporous structure with large pore size facilitates electrolyte diffusion and therefore can potentially enhance the capacitive performance when used as electrode.

**Figure 5:**
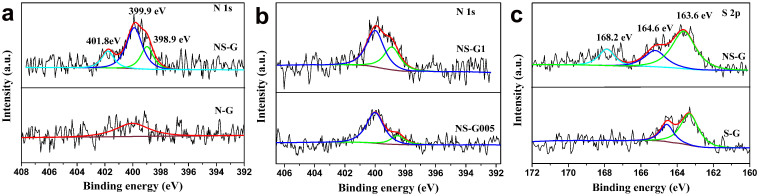
XPS spectra. (a) N 1s spectra of NS-G and N-G, (b) N 1s spectra of NS-G005 and NS-G1, (c) S 2p spectra of NS-G and S-G.

The doped samples were further investigated by high resolution XPS. As shown in Figure 5a, a strong N1s peak was observed for NS-G at about 400 eV, which can be deconvoluted into three components at 398.9 eV, 399.9 eV and 401.8 eV, corresponding to pyridinic, pyrrolic and graphitic nitrogen species, respectively^41^,^42^. It can be seen that pyrrolic nitrogen is the dominating species in NS-G, which is known to be able to contribute to pseudo capacitance^38^,^43^. Similarly, pyrrolic nitrogen groups were also the dominating nitrogen species in the co-doped samples synthesized from different cysteine to GO ratios (NS-G005 and NS-G1 in Figure 5b). It should be noted that only weak contributions were observed in the singly doped N-G sample (Figure 5a), indicating a much lower nitrogen concentration. As indicated by these results, it is likely that the amount of incorporated nitrogen is strongly correlated with that of sulfur.

Figure 5c shows the XP S 2p spectra of NS-G and S-G. The S 2p region of NS-G can be fitted with three peaks. The major contributions at binding energies of around 163.6 eV and 164.6 eV can be attributed to sulfur in C–S_n_–C (n = 1 or 2) and conjugated –C = S– bonds respectively^30^,^31^,^37^. The contribution at higher binding energy of around 168.2 eV can be assigned to the –SO_n_– species, which usually form at the edges of graphene^37^. In contrast, the oxide species were not detected in the S-G sample. The overall intensity of S 2p is higher in NS-G than in S-G, which again demonstrated the synergetic effect of N and S in the co-doped sample. Heteroatoms are typically incorporated at defect sites in carbon materials. Oxides species like –NOx and –SOx at edge sites are not thermally stable and can often be removed by intensive drying. Similarly, direct bonding between N and S is also unlikely after drying. However, they preferably exist in neighboring hexagonal rings. It was reported that O and N coexist and interact with each other in N-doped carbon nanotubes^44^. Especially, samples obtained from the liquid phase showed strong affinity between O and N. Similarly, S and N can have similar coordinated effect in the doped graphene samples. Although direct bonding between S and N might not be favored, they could be preferably incorporated into neighboring hexagonal rings. The presence of either of them could promote the embedding of another one in neighboring rings. Hence, the concentrations of both N and S were enhanced in the co-doped samples.

The surface atomic concentrations of C, O, S and N were derived from the corresponding peak areas of the XP spectra and the results are summarized in Table 2. The co-doped NS-G sample has a much higher surface atomic concentration of N (0.9 at.%) and S (0.3 at.%) than the singly doped samples (0.3 at.% N in N-G, 0.1 at.% S in S-G). Furthermore, the N and S elemental contents can be adjusted by varying the mass of cysteine. With the increase of weight percentage of cysteine, the N and S elemental contents were increased from 0.7 at% and 0.2 at% for NS-G005 to 1.3 at% and 3.2 at% for NS-G1, respectively (Figure S2 and Table 2).

## Discussion

Above-mentioned results demonstrate the unique features of the co-doped NS-G, including the high specific surface areas and pore volume, 3D porous structure, and high nitrogen and sulfur contents. These features strongly suggest that NS-G could have a better electrochemical performance than the singly doped samples.

**Figure 6:**
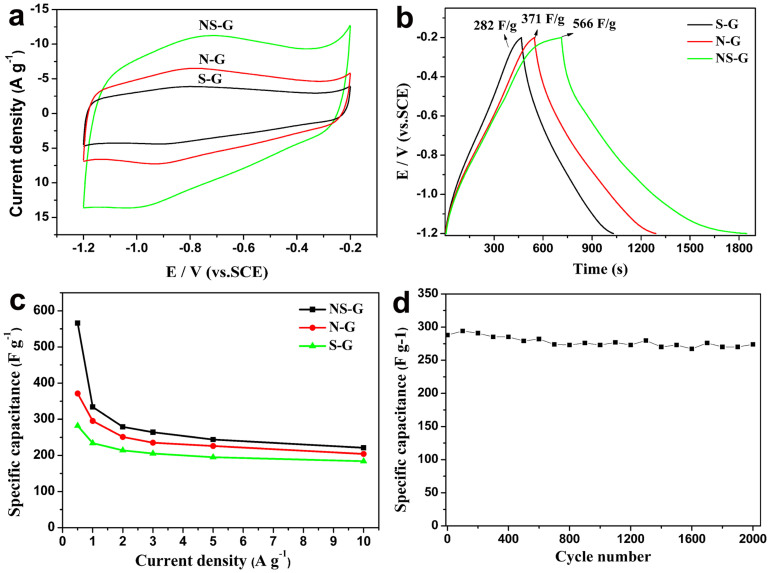
Electrochemical performance of the doped graphene. (a) CVs measured at 30 mV s^-1^ and (b) GCD curves measured at 0.5 A g^-1^ of NS-G, N-G and S-G samples. (c) The specific capacitance of NS-G, N-G and S-G at different current densities, (d) cycle performance of NS-G at the current density of 3.0 A g^−1^.

The doped graphene hydrogels were used as free-standing electrodes, and tested using cyclic voltammetry (CV) and galvanostatic charge-discharge (GCD) techniques with a three-electrode system. Figure 6a shows the CV curves of NS-G, N-G and S-G electrodes tested in 6.0 M KOH solution at a scan rate of 30 mV s^−1^. The voltammograms of the three samples show approximately rectangular shape, which are typical for double layer capacitors. Obviously, the NS-G has the highest capacitance in the three samples. Figure 6b shows the GCD curves measured at 0.5 A g^−1^. The curves show an approximately triangular form for the three samples, indicating the electric double-layer performance. The time of accomplishing a charge/discharge cycle is clearly longer for NS-G than that for N-G and S-G. The mass specific capacitances (Csc) calculated by using the equation Csc = (IΔt)/(mΔV) were 566, 282 and 371 F g^−1^ for NS-G, S-G and N-G, respectively. The Csc value of NS-G is higher than that of single doped graphene, RGO (277 F g^−1^ at 0.5 A g^−1^, see Figure S3) and those of other reported N, S doped carbon materials (Table S1). It should also be noted that the Csc value of NS-G (566 F g^−1^) is higher than the theoretical electrochemical double-layer capacitance of 520 F g^−1^^8^. Obviously, pseudocapacitance contributes to its electrochemical performance. Hence, the simultaneous incorporation of nitrogen and sulfur in the carbon framework effectively enhanced its capacitive performance.

It has been reported that pyridinic N in graphene layers results in the attraction of ions, and pyrrolic N has an enhancing effects on the capacitance due to its pseudocapacitive contributions^43^. The pyrrolic N can also improve the wettability of graphene in an aqueous electrolyte. Graphitic N can decrease the intrinsic resistance of carbon and improve electron transfer and the capacitive performance at high current loads^45^. In addition, the sulfur dopant species, such as thiophenic-like sulfur and sulfone, play significant roles in modifying the surface properties of carbon material^46^. The reversible redox reactions related to sulfone and sulfoxides species can also contribute to the pseudo capacitance of carbon materials^46^,^47^. The coordinated effect of S and N as discussed above can enhance the doping level of both elements. Additionally, the concentration of S and N could also be boosted by hydrogen bonds formed between S and N. The pyridinic and graphitic nitrogen can interact with H-S- groups and form N···H-S- bond. Similarly, hydrogen bond like N-H···S = might be formed too. As a whole, the graphitic N enhanced the electrical conductivity, and the high concentrations of pyrrolic nitrogen, thiophenic-like sulfur and sulfone enhanced the pseudocapacitance.

The calculated specific capacitance (Csc) at different current densities from 0.5 to 10 A g^−1^ is shown in Figure 6c (GCD curves of NS-G, N-G and S-G are shown in Figure 4S a–c). The specific capacitance of NS-G is higher than those of singly doped samples at all measured current densities, even though the SEM and BET results show that S-G and N-G display similar morphologies with NS-G. Hence, co-doping with N and S is more efficient in improving the electrochemical performance than single doping. Moreover, the cycling stability of NS-G was examined using galvanostatic charge/discharge cycling at a current density of 3 A g^−1^ for 2000 cycles (Figure 6d). The capacitance retention of 95% (274 F g^−1^) was obtained after 2000 cycles, which demonstrates the excellent cycling performance of the NS-G electrode.

**Figure 7:**
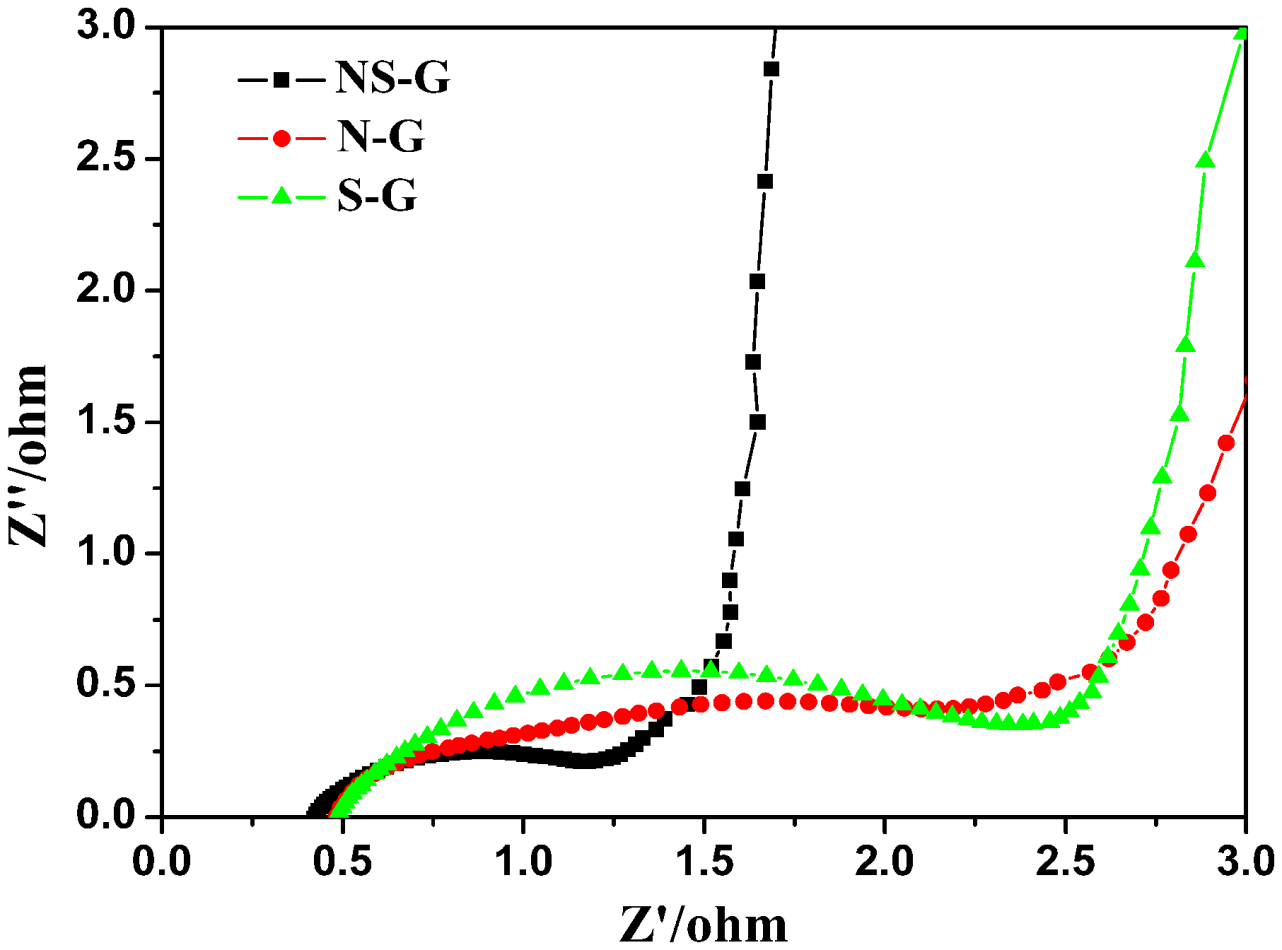
Nyquist plots of NS-G, N-G and S-G.

Electrochemical impedance spectroscopy (EIS) provides information on the internal resistance of the electrode material and the resistance between the electrode and electrolyte. The real part of resistance (Z′) measured is the ohmic resistance derived from the electrolyte and the contact between the electrode and the current collector. As shown in Figure 7, at very high frequency the internal resistance of the three doped samples is very low (below 0.6 Ω). Hence, the pyrrolic and other nitrogen species in NS-G did not cause a clear increase of resistance, which is different from surface oxygen groups, which often lead to the decrease of conductivity. In the range of medium-high frequencies an uncompleted semicircle loop can be observed. Obviously, a smallest semicircle is observed for NS-G. It is well accepted that the semicircle reflects the electrochemical reaction impedance of the electrode, and a smaller semicircle means lower charge transfer resistance^48^. Hence, the NS-G has the lowest charge transfer resistance, which may be related to the abundant heteroatom-doping. According to XPS results, a large amount of sulfur and nitrogen exists on the carbon surface, which increase the polarity of the carbon surface and facilitate its contact with aqueous electrolyte leading to decreased charge transfer resistance for NS-G^30^,^49^.

As to the samples synthesized from lower and higher cysteine to GO ratios, the specific capacitance at the current density of 0.5 A g^−1^ was determined to be 385 and 263 F g^−1^ for NS-G005 and NS-G1, respectively (Figure S4d and e). The electrode of NS-G showed a higher capacitance than NS-G1 although the latter sample possesses a larger amount of S. This result can be explained by the relatively lower specific surface areas of NS-G1 and NS-G005.

**Figure 8:**
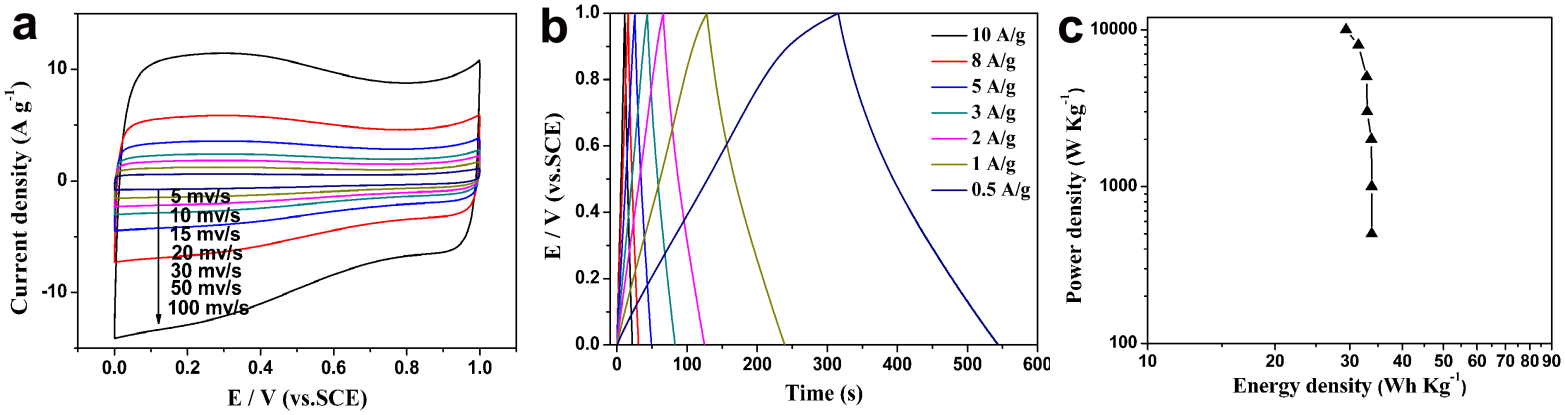
Electrochemical performance of the doped graphene. (a) CV and (b) GCD curves of NS-G electrode in a two-electrode system with different scan rates and current densities. (c) Ragone plot of NS-G.

Finally, the power density and energy density of NS-G was investigated by a symmetrical two-electrode system in 6 M KOH. The CV and GCD curves of NS-G at different scan rates and current densities are shown in Figure 8a and b, and the thus-derived Ragone plot is given in Figure 8c. At the scan rate of 1000 mv/s and 100 mv/s, the calculated specific capacitance is 121 F/g and 193 F/g, respectively (Figure S5 and Figure 8a). As shown in Figure 8b, the calculated specific capacitance is 212 F g^−1^ at the current density of 10 A g^−1^. The power densities were calculated using discharge curve of GCD. At a constant power density of 10 kW kg^−1^, the energy density obtained for NS-G was 29.4 Wh kg^−1^. These results indicate that NS-G is a promising electrode material for high performance supercapacitors. The cycling stability of NS-G was examined using galvanostatic charge/discharge cycling at a current density of 5 A g^−1^ for 2000 cycles (Figure S6) in a symmetrical two-electrode system. A capacitance retention of 93% (186 F g^−1^) was obtained after 2000 cycles.

In summary, nitrogen and sulfur co-doped hydrogels and aerogels were successfully synthesized through a hydrothermal method by using amino acid and GO as precursor. The obtained NS-G with a three-dimensional hierarchical structure containing both macropores and mesopores exhibited excellent mechanical stabilities. The simultaneous incorporation of S and N species with the presence of oxygen significantly modified the surface chemistry of carbon leading to considerably higher doping levels, although directly bonding between N and S is neither likely nor detected. Hence, the synergetic effect between N and S occurred through carbon atoms in neighboring hexagonal rings in a graphene sheet. When used as free-standing electrode in supercapacitors, the co-doped sample considerably outperformed the singly doped samples due to an enhanced pseudocapacitance and a lower charge transfer resistance. The specific capacitance of NS-G reached 566 F g^−1^ at 0.5 A g^−1^. The enhanced pseudocapacitance in the co-doped sample can be assigned to higher contents of pyrrolic N groups and S species.

## Methods

### Sample synthesis

Graphene oxide was prepared from graphite powder using a modified Hummers method^36^. The N- and S-doped graphene hydrogels were prepared by a hydrothermal assembly process. In a typical procedure, an aqueous dispersion of GO (2 mg ml^−1^, 30 ml) containing 0.05 mmol L-cysteine (C_3_H_7_NO_2_S the mass ratio of L-cysteine to GO is 0.1:1) was first treated by sonication for 10 min. The resulting stable suspension, sealed in a Telfon-lined autoclave, was hydrothermally treated at 160°C for 4 h. After that, the autoclave was naturally cooled to room temperature and the as-prepared NS-G hydrogel was removed from the autoclave and immersed in deionized water for seven days, in which deionized water was renewed once a day to remove any unreacted reagents. Finally, the NS-G hydrogel was freeze-dried overnight to obtain the aerogel. In addition to the L-cysteine to GO ratio of 0.1:1, a lower mass ratio of 0.05:1 and a higher ratio of 1:1 was investigated and the obtained samples are designated as NS-G005 and NS-G1, respectively. For comparison, N and S singly doped hydrogels (N-G and S-G) were prepared using the same procedure. L-alanine (C_3_H_7_NO_2_, 0.05 mmol) and 3-mercaptopropionic acid (C_3_H_6_O_2_S, 0.05 mmol) with 30 ml GO suspension (2 mg/ml) were employed for N-G and S-G, respectively. The hydrothermal synthesis was also performed without doping agent and the obtained sample is designated as RGO. The water content was determined by weighing the samples before and after drying.

### Characterization

All of the samples were characterized by scanning electron microscopy (SEM) using a Hitachi S-4800 system and transmission electron microscopy (TEM, JEM-2010F). X-ray diffraction (XRD) measurements were performed with a D8 Bruker diffractormeter with a Cu K_α_, (λ = 1.5418 Å) radiation. Raman spectra were recorded with a Bruker spectrometer with 532 nm laser, and X-ray photoelectron spectroscopy (XPS) measurements were carried out using a ESCALab220i-XL electron spectrometer (VG Scientific) using a 300W Al K_α_ radiation. Nitrogen physisorption was carried out at 77 K with an ASAP 2020 Physisorption Analyzer. The mechanical properties of the prepared hydrogels were investigated using a dynamic mechanical analyzer (DMA Q800).

### Electrochemical measurements

Cyclic voltammetry and galvanostatic charge/discharge of doped graphene electrodes were performed with a CHI660D electrochemical workstation with both two-electrode and three-electrode systems. The working electrodes were fabricated by the prepared hydrogels in the wet state without using any binder or conducting additives. A slice of the wet hydrogel was cut and blotted with a filter paper to remove excess water; then it was pressed onto a nickel foam sheet (3 × 1 cm) at 10 MPa for 30 s. The mass loading of active material on each current collector was about 2 mg/cm^2^ and the area of the prepared electrode is 1 cm^2^. In the three-electrode system, the graphene hydrogel samples were used as the working electrode, platinum foil as the counter electrode and standard Hg electrode as reference one, and 6.0 M KOH aqueous solution as electrolyte. CV and GCD test in the potential range of −1.2 to −0.2V was performed at different scan rate and current densities. For the electrochemical test in the two-electrode system, two slices of hydrogel with the same mass were pressed onto a nickel foam sheet (3 × 1 cm) at 10 MPa for 30 s and used as electrodes. The electrode contained approximately 2.0 mg/cm^2^ of active material. The two electrodes were separated by a filter paper, which was fully soaked with electrolyte. The potential range for CV and GCD tests was 0–1.0 V. Electrochemical impedance spectroscopy (EIS) measurements were carried out by applying an alternating current with the voltage of 5 mV over a frequency range from 0.01 Hz to 100 kHz. The gravimetric capacitance, C_CV_ (F g^−1^), was calculated from CVs using the following equation: C_CV_ = Σ|I|/2νmΔV, where Σ|I| is the area of the current (A) profile, m is the mass of active material in the electrode (g), ν is the scan rate (V/s) and ΔV is the potential window (V). The specific discharging capacitances were determined using the equation Csc = (IΔt)/(mΔV) from three-electrode measurement, and in the two-electrode system the energy density and power density were calculated using the equation E = 1/2 C_sc_ ΔV^2^ and P = E/Δt, respectively^50^.

## Supplementary Material

Supplementary Information Supplementary Information for Interaction between Nitrogen and Sulfur in Co-Doped Graphene and Synergetic Effect in Supercapacitor

## Figures and Tables

**Table 1:** Texture properties of doped graphene samples derived from nitrogen physisorption measurements

Samples	BET (m^2^ g^−1^)	Average pore size (nm)	Pore volume (cm^3^ g^−1^)
NS-G	305.2	14.8	0.61
N-G	361.1	5.6	0.23
S-G	322.4	9.2	0.41
NS-G005	285.1	9.6	0.25
NS-G1	156.9	10.2	0.57

**Table 2:** Surface composition of doped graphene samples derived from XPS studies

Samples	C at.%	O at.%	N at.%	S at.%
NS-G	85.1	13.7	0.9	0.3
N-G	85.5	14.2	0.3	—
S-G	86.5	13.4	—	0.1
NS-G005	84.2	14.9	0.7	0.2
NS-G1	83.2	12.3	1.3	3.2
