# Supplementary material for: Interaction between Nitrogen and Sulfur in Co-Doped Graphene and Synergetic Effect in Supercapacitor
Source: Sci Rep. 2015 Apr 16;5:9591. doi: 10.1038/srep09591 (PMC5381751; doi:10.1038/srep09591)
Supplement: Supplementary Information — for Interaction between Nitrogen and Sulfur in Co-Doped Graphene and Synergetic Effect in Supercapacitor [file srep09591-s1.pdf]

Supplementary Information

Interaction between Nitrogen and Sulfur in Co-Doped Graphene and Synergetic Effect in Supercapacitor

Tao Wang,[1] Lu-Xiang Wang,[1] Dong-Ling Wu,[1] Wei Xia*[2] & Dian-Zeng Jia*[1]

[1] Key Laboratory of Material and Technology for Clean Energy, Ministry of Education, Key Laboratory of Advanced Functional Materials, Autonomous Region, Institute of Applied Chemistry, Xinjiang University, 830046 Xinjiang, P. R. China.

[2] Laboratory of Industrial Chemistry, Ruhr-University Bochum, 44780 Bochum, Germany.

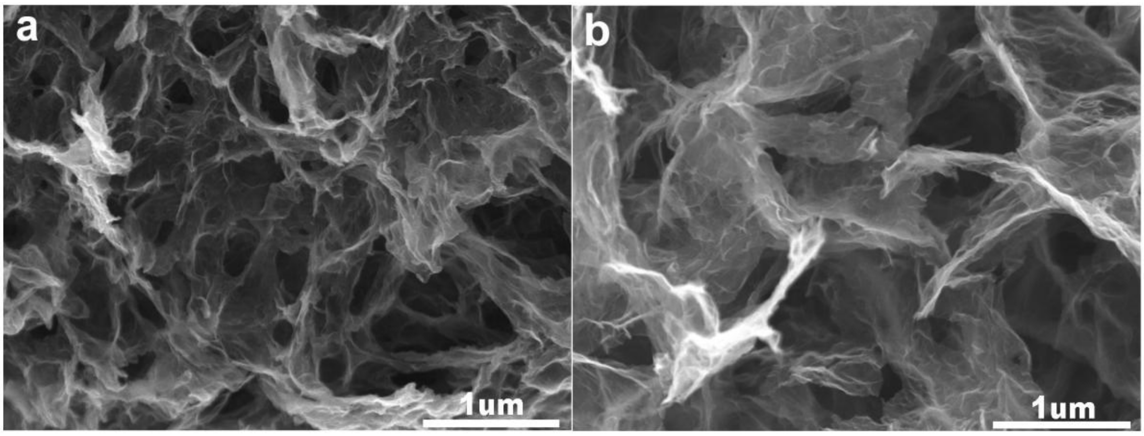

Figure S1 SEM images of the as-prepared (a) NS-G005 and (b) NS-G1.

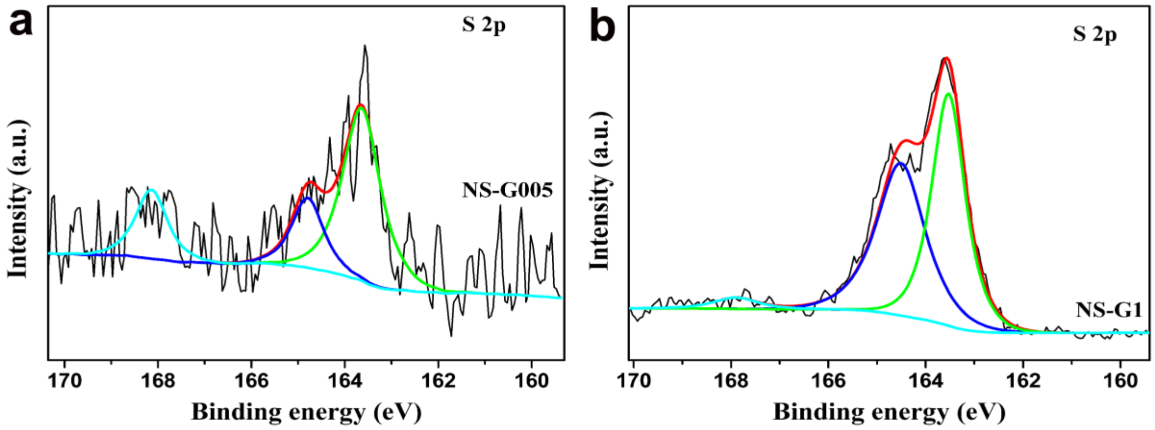

Figure S2 S 2p region spectra of (a) NS-G005 and (b) NS-G1.

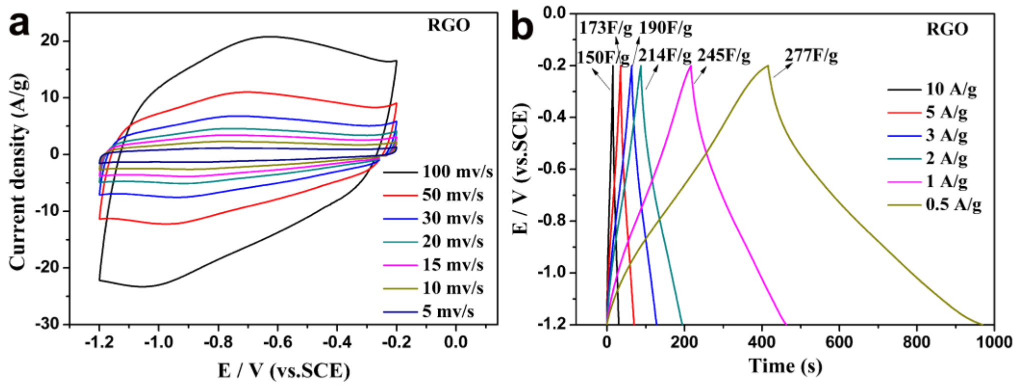

Figure S3 (a) CV curves for rGO measured at different scan rates, (b) GCD curves for rGO at different currents

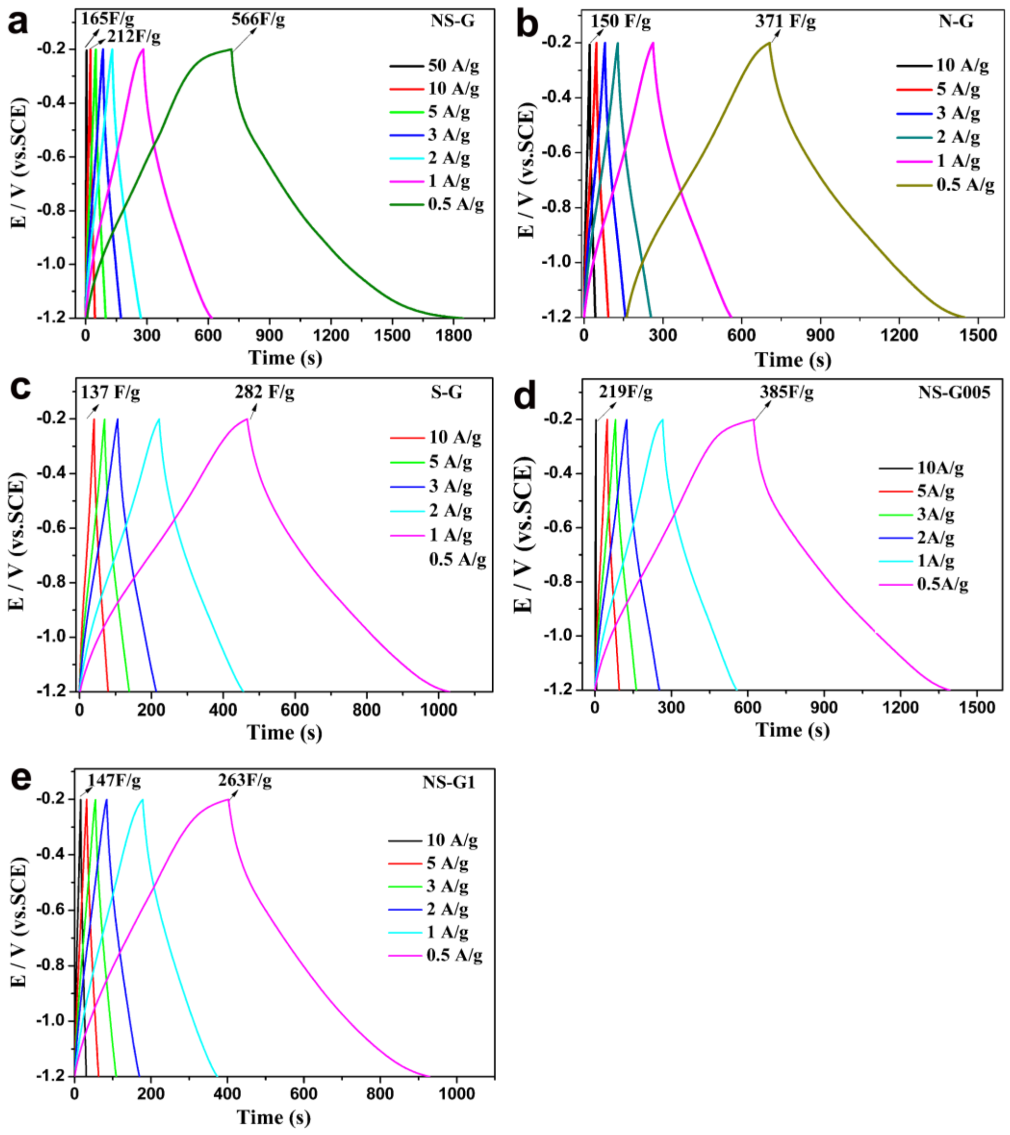

Figure S4 GCD curves for (a) NS-G, (b) N-G, (c) S-G, (d) NS-G005 (e) and NS-G1 obtained at current densities of 0.5-10 A g^{-1}.

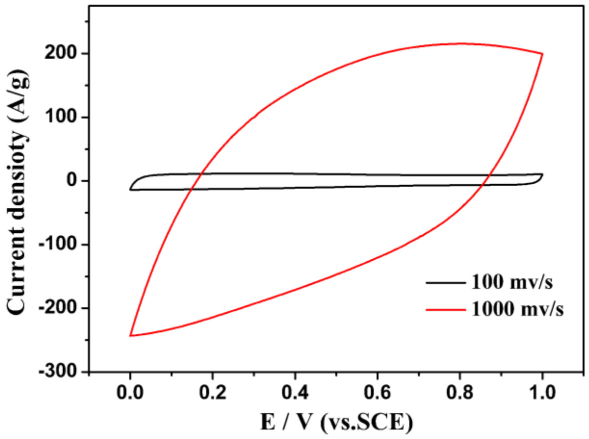

Figure S5 CV curves of NS-G measured at the scan rate of 1000mv/s and 100mv/s respectively.

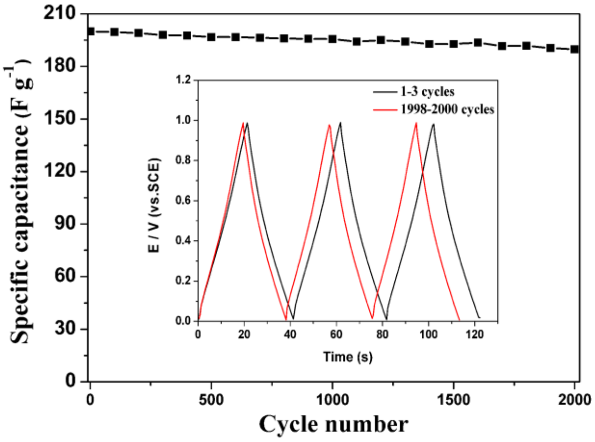

Figure S6 Variations of specific capacitance versus cycle number of NS-G measured at a current density of 5 A g^{-1} within the potential range from 0 to 1.0 V in the symmetrical two-electrode system. The inset shows the charge–discharge curves between 1–3 and 1998–2000 cycles for NS-G.

Table S1 Specific capacitance of N, S co-doped carbon materials.

Materials	Electrolyte	Current density	Electrode system	Capacitance	Ref.
N, S containing carbon	1 M H_2SO_4	0.05 A g^{-1}	Three	138.8 F g^{-1}	S1
N, S co-doped mesoporous carbon	2M KOH	1 A g^{-1}	Three	320 F g^{-1}	S2
N, S doped activated hydrothermal carbons	6M KOH	0.25 A g^{-1}	Three	264 F g^{-1}	S3
N, S co-doped mesoporous carbon	2M KOH	1 A g^{-1}	Three	180 F g^{-1}	S4
N, S co-doped graphene	6M KOH	1A g^{-1}<br>0.5 A g^{-1}	Three<br>Three	334 F g^{-1}<br>566 F g^{-1}	Present work

Supplementary References

S1. Tsubota, T., Takenaka, K., Murakami, N. & Ohno, T. Performance of nitrogen- and sulfur-containing carbon material derived from thiourea and formaldehyde as electrochemical capacitor. *J. Power Sources* **196**, 10455-10460 (2011).

S2. Zhang, D. *et al*. Synthesis of nitrogen-and sulfur-codoped 3D cubic-ordered mesoporous carbon with superior performance in supercapacitors. *ACS Appl. Mater. Interfaces* **6**, 2657-2665 (2014).

S3. Si, W. J. *et al*. Tunable N-doped or dual N, S-doped activated hydrothermal carbonsderived from human hair and glucose for supercapacitor applications. *Electrochim. Acta* **107**, 397-405 (2013).

S4. Zhang, D. Y. *et al*. Nitrogen and sulfur co-doped ordered mesoporous carbon with enhanced electrochemical capacitance performance. *J. Mater. Chem. A* **1**, 7584-7591 (2013).
